# Incitement, genocide, genocidal terror, and the upstream role of indoctrination: can epidemiologic models predict and prevent?

**DOI:** 10.1186/s40985-018-0106-7

**Published:** 2018-10-22

**Authors:** Elihu D. Richter, Dror Kris Markus, Casey Tait

**Affiliations:** 1 Jerusalem Center for Genocide Prevention, Jerusalem, Israel; 2 School of Public Health and Community Medicine, The Hebrew University - Hadassah Medical Center, Ein Karem, POB 12272, 9112001 Jerusalem, Israel; 3 Regent University, Virginia Beach, USA

**Keywords:** Epidemiology of genocide and genocidal terror, Indoctrination, Incitement

## Abstract

We apply the models and tools of epidemiology and public health to propose a unified field theory showing the role of ideologies, indoctrination, and incitement, in genocide, genocidal terror, and terror by groups or individuals. We examine the effects of indoctrination and incitement as exposures and risks in relation to genocide and genocidal terror. Incitement has been recognized as a trigger to these outcomes but indoctrination is upstream to incitement. Population-wide exposure to indoctrination increases susceptibility to the effects of incitement. These relationships have been seen in all major genocides and genocidal terror in the late twentieth and twenty-first centuries. There is some insight into the relationship between ideology, incitement, and genocidal acts of violence from the so-called localized genocides in Bosnia, Rwanda, Darfur, Syria, and most recently, among the Rohingya in Myanmar. There is a need to recognize the upstream role of ideologies of hate in order to determine the degree to which indoctrination posed, and continues to pose, a contributing factor. Epidemiologic models, such as the iceberg model of exposure and disease and the concept of “sick individuals” and “sick populations,” guide our understanding of the content and spread of indoctrination and incitement and can provide essential insights for prevention. The hateful indoctrination and ideologies behind genocidal violence must be countered and replaced by positive ideologies and role models that emphasize respect for life and human dignity for all.

## Background

The ghastly consequences of genocide, genocidal terror, and terror are man-made. Therefore, their prevention should be man-made. Our premise is that prediction and prevention can counter hateful indoctrination that leads to incitement, willful acts of violence, and ultimately, genocide. Incitement, genocidal terror, and genocide result from human choice and bystander indifference and constitute an extreme assault on human life—the basic human right [1]. Therefore, we have an obligation to counter upstream ideologies that indoctrinate hate and incitement. Rabbi Abraham Heschel said, “Auschwitz was built not with stones, but words” [2]. The world can eradicate genocide and genocidal terror by being vigilant and reinforcing a policy of zero tolerance for indoctrination of hateful ideologies and incitement to genocide.

We briefly review the existing predictive models of genocide. These models mention the role of incitement as a precursor indicator, predictor, and catalyst of violent action. However, these models beg the question of what leads to the incitement. We propose recognizing ideological indoctrination as being upstream to incitement and genocidal violence—forced expulsion, torture, rape, killings, mass murder, beheadings, mutilation, executions, starvation, and other crimes against humanity, and therefore, requiring examination. We propose a unified field theory to explain this relationship. These models are meant to be predictive; thus, they state the case for preventive measures.

There is a long-established perception that incitement can lead to genocide. This has been spelled out in the United Nations (UN) Convention on the Prevention and Punishment of the Crime of Genocide and in the Rome Statute of the International Criminal Court (ICC) [3, 4]. The first prosecution of a war criminal based on incitement was the conviction of Julius Streicher in the Nuremberg trials. Streicher was executed for crimes related to the vicious anti-Semitic incitement in his newspaper, *Der Sturmer* [5]. This conviction represents the first application of the principle of prosecuting an upstream driver to genocide in international criminal law.

More recently, following the Rwandan genocide, the ICC prosecuted a number of sources of incitement [6]. Gordon presents a full discussion of legal responses to Rwandan incitement [7]. The deportation from Canada and subsequent trial of Leon Mugesera, a Rwandan politician who gave an inflammatory speech 2 years before the genocide carried out in 1994, sets a new precedent [8]. This indictment established the principle that incitement to genocide, even without a direct relationship to acts of murder and violence, is itself a crime against humanity [9]. It is generally accepted, therefore, that incitement leads to the acts of violence cited above. In this paper, we ask: what leads to the incitement?

### Indoctrination and word pollution

We build upon previous work in which the case is made for using epidemiologic models to show the relationship between incitement and genocide [10]. Word pollution—the motifs, language, and images of incitement—can be defined as an exposure leading to the wide range of adverse outcomes in genocide: mass murder, torture, rape, forced expulsion, and other crimes against humanity. We focus on the need to recognize the indoctrination of specific ideological perspectives as a necessary precursor and predictor to both widespread word pollution and incitement.

In the discussion of word pollution, incitement, and indoctrination, the concept of propaganda comes to mind. It is important to note that propaganda, a comprehensive concept in itself, refers to the methods of persuading and influencing behavior of mass audiences [11]. Jowett and O’Donnell define propaganda as the “deliberate, systematic attempt to shape perceptions... and direct behavior to achieve a response” based on the “desired intent of the propagandist” [12]. The role of propaganda in creating “climate[s]” of genocide has been recognized by international courts in a number of cases throughout history and prosecuted as a crime [13]. Propaganda is one method of word pollution spread. These techniques can be found among messages in both incitement and indoctrination.

The term word pollution projects the concept that incitement is a hazardous exposure, with sources, paths of exposure, and adverse effects among susceptible populations that are analogous to air pollution. We can examine the intensity and frequency of such exposures and their source, modes of spread, and target populations (in keeping with classic epidemiologic models). Such models can help us define strategies for predicting the spread of word pollution and evaluating interventions. As epidemiologists, our work is disciplined by asking the basic questions: who, what, where, when, how, and so what. Where possible, we quantify the exposures and effects. By constructing timelines, maps, and graphs, we can better design interventions to prevent the horrors of genocide, genocidal terror, and terror.

### Key terms and definitions

The UN Convention on the Prevention and Punishment of the Crime of Genocide defines genocide as acts committed with the intent of destroying, in whole or in part, a group based on its national, ethnic, racial, or religious origins (other definitions include political view, gender, and orientation). Genocide includes killing, serious bodily or mental harm, expulsion, impairing reproduction or transfer of young, and making conditions of life impossible [3, 14]. Genocidal terror is defined as any violence directed at a group singled out by its national, ethnic, racial, religious, or political origins [15, 16]. We adopt the United States (US) Government’s definition of terrorism: premeditated, politically motivated violence perpetrated against noncombatant targets by sub-national groups or clandestine agents, usually intended to influence an audience [17].

Incitement does not pertain to a belief system; it consists of speech, writing, and images whose purpose is to rouse individuals or groups to violent action [18]. This definition includes what we refer to as the “5 Ds”—dehumanization, demonization, delegitimization, disinformation, and the denial of past atrocities perpetrated against the target. Incitement now includes glorification of terror and threats, issues of special concern today (see Table 1) [14]. These forms of incitement can carry enormous weight, especially when initiated or endorsed by persons of authority. Incitement alone does not necessarily lead to the perpetration of acts of violence in genocide and genocidal terror. The impression is that those carrying out the acts were likely already programmed or conditioned by prior indoctrination or by much repeated incitement.

**Table 1:** The “5 Ds +” of incitement

Term	Definition
Dehumanization	Dehumanization is used by perpetrators to evoke feelings of loathing, contempt, and revulsion, often by comparing or identifying the target with nonhuman species or diseases.
Demonization	Blaming the target for the perpetrators personal misfortunes or those of his/her group and/or provoking feelings of fear towards a specific group.
Delegitimization	Denying the existence or history of the other group, and/or accusing the target of extreme criminal acts.
Disinformation	Presenting false or partial information with the intent to malign.
Denial	Negating historical facts or denying past atrocities.
Threats	Statements of intent to inflict pain, injury, damage, or other hostile action on groups or individuals.
Glorification of terror	Invoking well-known perpetrators of genocidal violence as role models (such as the memorializing “martyrs” or financial compensation for families of “martyrs” or terrorists)

A classical definition of indoctrination refers to “unethical influencing” in education and the passing on of beliefs without leaving room for critical thinking and assessment [19]. Indoctrination includes the systematic transmission of values or beliefs representing the core ethos of any given group. It is often comprehensive, intergenerational, and deeply embedded within society. Indoctrination instills and reinforces fundamental beliefs and messages, which program audiences to be receptive to explicit instructions or actions based on that ethos—or in the case of hateful ideologies, respondent to the messages and motifs of incitement.

The themes of hateful indoctrination are often based on ideologies of racial or religious supremacy, chauvinism, and demonization of the other. Ideology is at the basis of programs and systems that instruct, organize, train, persuade, intimidate, and coerce populations. Indoctrination presupposes a framework of ideologies, movements, and organizations supporting, instructing, directing, intimidating, and inspiring action—often in a coercive manner that is either authoritarian or totalitarian. We see systems of indoctrination producing conditioned populations that can be easily mobilized by exposure to the messages and motifs of incitement.

### Violent totalitarianism under the title of jihad

Recent reviews of genocides focus on the major killers of the twentieth century—Nazism and Communism (including its Maoist variants)—as well as more recent cases such as Bosnia (death toll estimated at 100,000), Rwanda (between 500,000 to one million), Darfur (estimated at 300,000), Syria (estimated at 500,000), and Myanmar (hundreds killed and over 140,000 displaced) [20].

Of interest to our case study is the rise of certain streams of Islamic ideology. The collapse of the Ottoman Empire and the advent of the modern nation-state influenced the spread of Muslim religious political groups and movements (notably, the Muslim Brotherhood) whose primary concern was to address the cultural identity of Islam and how Sharia law would manifest in the contemporary world [21]. “Sharia” or Islamic law, derived from the Quran and the traditions of the Hadith, is the holistic body of rules and teaching which regulates public and private life, governing every aspect of Muslims’ relationship between family, society, and nation. Sharia is the basis of moral, theological, and legal motivation and justification for all aspects of individual and community life [21–23]. There is a wide variety of adherence and interpretation of Sharia among Muslim groups worldwide, and in particular concerning the tenet of jihad, expression of which can be offensive or defensive [21]. Central to the concept of jihad and Sharia is the universal and perpetual obligation of every devout Muslim to “wage jihad,” whether by means of peaceful struggle or violent resistance, against the Kafir (infidel or unbeliever) until they either submit or convert to Islam [21, 22].

We focus on the current global challenge of violent totalitarian indoctrination systems and motifs of incitement under the title of jihad. We acknowledge the challenges in defining the term jihad, as a classical definition can imply struggle against one’s own soul’s evil inclinations, whereas military jihad means fighting to facilitate Islam’s spread or defend its realm against its enemies. In this latter capacity, its radical vindicators may sometimes call for the total or partial eradication of non-Muslim groups, as well as of Muslims not of the “correct” belief system, as articulated recently by ISIS (or Daesh)—Islamic State in Iraq and Syria. We call for the need to recognize the ideology espoused by this particular stream of jihadist totalitarianism in its various manifestations as global in outreach like Nazism and Communism were in the height of their power. These ideological systems can become genocidal when totalitarian regimes implement the 5 Ds of incitement as well as glorification of terror.

The case study in this paper presents specific examples pertaining to jihadist totalitarianism, which presently represents the most formidable geopolitical threat, as acknowledged by the US State Department, having produced war, genocide, and terror in the Middle East, as well as other parts of Asia and Africa, has contributed to the spread of terror in Europe, and has increased the danger posed by Islamic countries possessing nuclear capabilities [24, 25]. The various streams of jihadist ideology have used indoctrination and incitement to recruit and motivate followers and to promote terror and genocidal agendas. Jihadist totalitarianism used to have traditional political boundaries, but with the digital revolution and social media, it can now reach everyone, everywhere, all the time. The pandemic spread of extremist jihadist messages and motifs has reached more than one billion people in multiple continents, led to the murder of millions (the vast majority of victims being themselves Muslim), and continues to be a force [26–28]. The horrific tolls state the case to develop preventive measures and interventions to stop the spread of the ideology and indoctrination systems.

## Themes and thrusts of our review

We devote special attention to three recent important publications offering predictive models of genocide and mass atrocities: Verdeja (2016), Maynard and Benesch (2016), and the UN Framework of Analysis for Atrocity Crimes (2014) [29–31]. These reviews consider legal and historical aspects of genocide and mass atrocities and offer predictive models from case studies. Verdeja reviews a number of predictive models for genocide and mass atrocities and distinguishes between risk assessment (RA) and early warning (EW) models. Risk assessment is based on long-term structural variables of a country or area. Early warning examines factors that can trigger immediate acts of violence. Maynard and Benesch develop a model integrating “dangerous speech” (incitement) and the ideologies behind them. The UN Framework lists a number of risk factors with subsequent indicators that can be used to predict future mass atrocities.

What is noteworthy is that these papers pay little attention to the upstream role of indoctrination leading to the perpetration of violence. Verdeja includes “hate media,” “public rallies,” and “popular mobilization” among the early warning indicators of genocide prediction models [29]. Maynard and Benesch note the “ideological context” behind violent acts. Indoctrination is the missing piece connecting the two. The UN statement includes supremacist ideologies, inflammatory rhetoric, and hate speech as indicators. However, not once is the term “indoctrination” used.

This is particularly telling, as incitement alone is not enough to predict violence. In his empirical study to test the causal effects of radio incitement on acts of violence in Rwanda, Straus was unable to prove a direct relationship between incitement and violence [32]. His analysis demonstrates that incitement alone is not the primary cause of killing, mass murder, beheadings, rape, torture, forced expulsion, and other crimes against humanity. Rather, he says, incitement is effective only in the event of preconditions. We propose that these preconditions are systems of indoctrination. This statement seems intuitive. Simply hearing hate speech on the radio will not automatically send every individual off to begin killing. Only those who have been sensitized to such messages over time will actually act upon them. Thus, a predictive model must include such upstream factors.

Recent literature on terrorism lacks sufficient discussion of the critical role of indoctrination. Pardo has provided a comprehensive analysis of anti-Semitic motifs replete in school textbooks utilized in both Iran and by the Palestinian Authority (PA) [33]. Baker describes cases of anti-Semitic incitement in mass media, government speeches, and educational systems [34]. In each of these cases, we see the terms incitement and indoctrination being used interchangeably. By distinguishing between these terms, we can develop better countermeasures to such phenomena.

How are indoctrination and incitement related? In essence, indoctrination is the hardware and incitement is the software. Ideologies are systematically constructed and communicated through speech, written materials, and messages and motifs delivered by influential leaders and figures of authority (top-down messages). Ideologies can be produced by individuals; yet authentically creative thought is generally stymied in favor of assuming prominent ideas from society or groups. Most ideological development is both driven by and representative of a relative minority [35]. School books and other official sources provide the indoctrination whereas social media organizes and promotes the incitement [36]. Repetitive incitement can harden into indoctrination and vice versa. An example of these relationships is the way ISIS’s viral videos have assumed an enduring life of their own. Such videos can indoctrinate anyone—sitting alone in front of their computer screens anywhere in the world—to the point of violent action. But they are most effective on those who are already sensitized.

### Epidemiologic models

We suggest that epidemiologic models can be used to better understand, predict, and prevent today’s pandemic spread of hateful indoctrination and incitement and their conversion to violent action. Epidemiology focuses on the distribution and determinants of infectious diseases, the source and spread of non-communicable diseases, and social phenomena (e.g., crime, road injuries, buying and the spread of hate content via the Internet) [15]. Quantitative risk assessment examines the relationship between exposure to a hazard and outcomes and, based on such models, has been used “proactively” to “support regulatory decisions” and shape policy [15]. Researchers identify sources, vulnerable populations, and the determinants of outbreaks. Epidemiologists analyze data and develop predictive models that can be used to suggest relevant interventions and preventative measures. Understanding and quantifying cause-effect relationships between the sources and their spread is both a strength and a challenge of epidemiologic models [15].

Such models need not be limited to the study of disease. Social behavior can mimic the spread of biological organisms (think of a “viral” video spreading across social media) [15]. Social scientists can take social phenomena and apply epidemiologic models to track and measure their spread. We propose taking models used to track the health effects of air pollution to track and measure the effects of word pollution. Such pollution consists of incitement and, more importantly, the role of indoctrination in producing such incitement. The messages and motifs of word pollution are without borders, embedded in cyberspace, with universal reach. Therefore, there is a need to gather empirical data concerning factors that contribute to individual and population resistance to these motifs and messages and to propose strategies that counter the negative effects of such ideologies.

By defining hateful motifs and messages as an exposure, we can track and explore exposure-effect relationships, track source, intensity, and frequency, and monitor time frames. We can also examine the social circumstances that influence the effects of varying motifs and messages, which can provide insight into which interventions might be effective in preventing the spread of hateful indoctrination and incitement. Stein and Richter propose several relevant epidemiologic models and appendices in their 2012 monograph and present empirical data and ecologic correlations which state the case for prediction, precaution, and prevention [15].

While other epidemiologic models such as social determinants, ecologic, and complex systems models might offer explanations and insights for preventive efforts, we focus on the infectious disease model based on the premise that word pollution most closely resembles the spread and effect of community-wide air pollution–as has been extensively studied in Beijing and Los Angeles [37, 38].

#### The Iceberg model

**Fig. 1:**
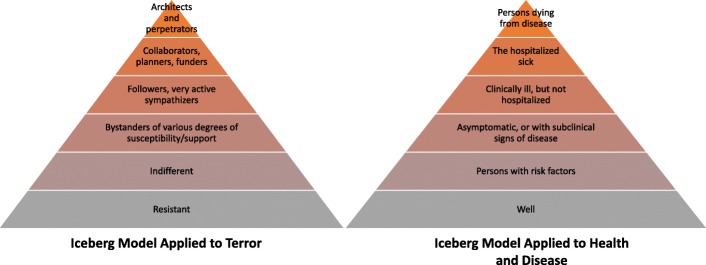
Iceberg model of disease applied to terror

The “Iceberg model” of disease shows how, for every hospitalized patient suffering severe disease symptoms, thousands more “walking sick” exist with subclinical disease who should be targeted for intervention and many more are at risk due to background effects or exposures [39]. Applied to terror, the Iceberg model expresses the concept that perpetrators are drawn from a larger population-wide pool of increasing degrees of risk and participation as sympathizers, endorsers, followers, recruits, and ultimately participants or perpetrators (see Fig. 1, Iceberg model of disease applied to terror). For example, supporters of an ideology that glorifies acts of genocide or genocidal terror may themselves be passive. However, with repeated stimuli, these supporters can become active participants.

Perpetrators are at the top of the epidemiologic iceberg, either as members of groups or as individuals. These perpetrators come from a larger pool of already sensitized individuals and groups and can be activated by triggers towards the pinnacle of a multistage process. The model is dynamic. Individuals and groups move up and down in relation to exposure to incitement and susceptibility to its effects. At any given moment, there are segments of the population at each of the levels. With enough exposure to the aggravating agents (in the case of word pollution—indoctrination), members of a lower rung in the pyramid can rise up to higher levels.

Intervention or deterrence directed at removing or neutralizing the actions of those at the top of the iceberg by itself cannot be expected to remove the source of the influence directed at the entire population. It is futile to simply hunt for terrorists when radical ideologies prevail in entire populations. The best example is that the assassination of Bin Laden did not kill off Al-Qaeda. Prevention or eradication of genocidal terror requires more than tactical defeats or punitive measures against the perpetrators of violence. Population-wide counter-strategies must be implemented.

#### Geoffrey Rose model

A second model is the Geoffrey Rose model [40]. Rose, an epidemiologist, coined the concepts “sick populations and sick individuals” and “the mean determines the range” [41]. These concepts indicate how population-wide interventions to remove hazardous exposures are more effective at reducing the total number of diseased individuals than interventions treating a smaller number of sick individuals at the peak of the iceberg. The implication is that population-wide measures can shift the entire range of the population distribution towards greater health. For example, reducing the population-wide exposure to salt in our diets is a population base approach to augment case finding and treatment of individuals with high blood pressure.

We can apply the Rose model to indoctrination. Population-wide exposure to indoctrination that leads to incitement, genocide, and genocidal terror pushes the mean towards the “sick group”—the perpetrators of violence (metaphorically “heating-up heads”) [6]. The implication is that population-wide countermeasures that target susceptible groups, those at high risk from exposure, will be more effective than targeting sick individuals. We are not aware of this concept having been applied to the epidemiology of genocidal indoctrination and incitement unto terror as of yet.

We recognize that there may be times that the range determines the mean. High-risk individuals may themselves have an influence on the rest of the population by serving as heroic role models or passing on incitement to others. The people at the tails can pull the entire population to opposing directions.

This concept is relevant to both negative and positive deviance and their impact on behavior. By reducing population-wide exposure to hateful indoctrination and introducing measures that strengthen positive deviance, the mean and distribution of susceptible groups can be shifted away from the so-called sick group towards greater health.

#### Positive deviance

Positive deviance refers to the observation that, in every population group, there are individuals at the positive end of the spectrum. These outliers are worthy of emulation and should be strengthened. For example, in any cases of mass violence, there are always individuals who defiantly refuse to “join the crowd” [42]. They resist both official orders and social trends.

The basic concepts of positive deviance come from the classic works of Milgram. He found that there are always individuals who disobey orders or social pressures that contradict their personal judgment or values. In his study on obedience, Milgram addressed the participants who refused instructions in the simulated shock experiment [43]. In public health, these are the individuals who resist social pressure to smoke cigarettes, use drugs, or engage in other high-risk or socially harmful behaviors.

Documentarian Yoav Shamir explored this concept of positive deviance in his documentary “10%, What Makes a Hero?” [44]. He studied people who resist societal trends in accordance with their personal convictions. The challenge for those concerned with genocide and genocidal terror is to identify individuals that are resistant to the effects of exposure to hate indoctrination and incitement and to have their examples serve as multipliers. The fact that such individuals exist in every social group offers the potential for prevention.

Examples of positive deviance are the Righteous Gentiles from the Holocaust, Mohammed Dajani and the Palestinian Wasatia Movement, the Quilliam Organization in the UK, Dr. Qanta A. Ahmed, and Dr. Zuhdi Jasser, a Syrian American whose Center for Islamic Pluralism counters motifs of Jihadist totalitarianism with positive messages—to name a few [45]. Their examples should be lauded and emulated as they help to “melt the bottom of the pyramid” of the iceberg.

#### Susceptible groups

Another concept from epidemiology is the identification of “susceptible groups.” These are population groups that are particularly vulnerable to the effects of health hazards. One example is children. Epidemiologists recognize that children are generally more vulnerable to toxic agents such as lead.

This concept is true for indoctrination and incitement as well, as children are particularly susceptible to indoctrination via textbooks, television, youth groups, and messages delivered by authority figures. Burdman has extensively explored the concepts of indoctrination mechanisms used on children [46]. Youth is one factor in susceptibility.

Burdman explains how certain cultures may be more susceptible than others. Indoctrination messages are “more readily accepted” by collectivist authoritarian societies where “obedience is the rule” and a “quasi-Pavlovian response” is the norm [47]. The challenge is to find out what reverses such susceptibility. Richter and Stein’s epidemiologic monograph gives further models and examples of this phenomenon [48]. The enduring effects of indoctrination among susceptible populations are noteworthy.

Many exposures and effects can be intergenerational when the source of transmission is persistent. This concept is relevant for understanding the contagious spread and multiplying effects of cradle-to-grave exposure to indoctrination and incitement. This principle can be exploited for good or for evil (e.g., educational systems that promote human rights vs. perpetuating intergenerational transmission of hate).

Research exploring neuropsychological structures reveals that the socio-physiological effects of indoctrination and incitement can lead to conditioning, patterning, habituation, and normalization (personal communication of unpublished observations: Trevor Davis). Thus, children raised in educational systems that indoctrinate towards hatred and violence are more likely to pass on such values to their progeny as well. Such intergenerational transmitted ideologies can be extremely resistant to change as they multiply and merge to become part of the core beliefs and values of society.

### The fallacy of the lone wolf

Based on the concepts and epidemiologic models presented, it is clear that the phenomenon of the “lone wolf” is problematic and possibly non-existent. So-called self-radicalization can only occur with prior exposure to the motifs of radical hate ideologies. Lone wolves emerge from populations and sub-groups already susceptible to background exposure. These individuals have been sensitized and can easily be triggered to commit acts of terror. Such sub-groups are susceptible to the slightest trigger impulses (e.g., highly charged or politicized words or concepts). In this sense, they appear to be lone wolves, but, in reality, they have been highly sensitized to extreme motifs and messages by specific ideological indoctrination.

Lone wolves represent the individuals at the “sick” end of the curve in Geoffrey Rose’s model, as opposed to those at the “healthy” end who exemplify positive deviance. We propose a two-stage model for background exposure and trigger effects. The first stage involves everyday background exposure, and the second stage relates to trigger effects. We suggest, for example, the use of the term “jihad” as a trigger word for groups already sensitized by previous background exposure to a complex set of messages and motifs of fear, hate, and threat. Such trigger effects only occur among those with strong background exposure similar to classic initiation of carcinogenesis.

### Case study

For this study, we focus on the pandemic spread of violent versions of Islamic theocratic intergenerational indoctrination and incitement based on the aforementioned extremist interpretation of traditional jihad. Like population-wide exposure to deadly toxins, the spread of evil motifs espoused by this extremist ideology inspire and motivate millions and recognize no conventional geographic or political boundaries. Today, the threats are directed against Jews, Christians, moderate Muslims, Kurds, Yezidis, and minorities throughout the Middle East [36]. This extremist version of jihad bears parallels to Nazism in the sense that it is totalistic, authoritarian, and harnesses state-sanctioned power to coerce and promote its Orwellian aims. Similar to Communism, this stream of extremist jihad is absolutist in its goals, promotes perpetual war with no moral limits (in contrast with the rigid moral limits of traditional jihad in Islamic doctrine), and permits conquest of all who are not considered “true believers.”

The major streams of such indoctrination and incitement are as follows: the Shiite theocratic regime in Iran; the Wahhabi sect in Saudi Arabia (at least, until recently); some prominent Sunni religious leaders whose theological base is Al-Azhar University; and various extensions that have drawn from the ideology of the Muslim brotherhood. Extreme jihadist ideologies are most readily identifiable in recognized terrorist organizations such as Hezbollah, Hamas, Boko Haram, and ISIS, which is the most gruesome and radical expression of Sunni jihadist totalitarianism [49].

Common to all these extremist religious streams is the demonization of Israel and attributing to Zionism the traditional motifs of Jew hatred. They promote intergenerational transmission of these motifs and messages, which are embedded deep in the educational systems of schools and mosques and are population-wide in their reach. There are formidable challenges to changing the beliefs and mindsets produced by these ideologies, which are systemically woven into the very fabric of Muslim societies, especially where theocratic rule prevails. An alarming example of this ideological indoctrination is the fact that Mein Kampf—itself a source of vile incitement—has become one of the most popular books sold in the Muslim world [50]. Its circulation is a measure of how deep the messages and motifs of genocidal ideologies have penetrated into that world [51].

For this case study, we consider two major categories of indoctrination and incitement: top-down dogmatic messages by figures of authority and educational systems—both of which represent powerful, pervasive, and population-wide forms of intergenerational ideological indoctrination.

#### Education: the fundamental source of a society’s ideological training

“The preeminent feature of a nation-state is its system of education” [52]. Textbooks, among other state-sanctioned means of education, are one of the most important channels for indoctrination. The messages found in school books reflect the core values, morals, and beliefs a given society wishes to promote. School books are especially effective as children are a captive and often vulnerable audience. Specific messages, both positive and negative, are delivered to the young even in the form of nursery rhymes and early formative education, the effects of which are enduring and intergenerational. School books are used in a controlled environment—the classroom—and represent ideology which symbolizes the intent of an organized authority. Our hypothesis is that populations with lifetime, cradle-to-grave exposure to messages and motifs that incite against others are more likely to engage in genocidal violence than populations not so exposed—and this risk increases with the intensity and frequency of exposure.

#### Top-down authoritative indoctrination

Ideas carry long-lasting power, especially when promulgated through authoritative systems. Totalitarian jihadist ideology combined with strong state power can be extremely dangerous. Powerful authority figures indoctrinate hateful ideology for generations when they glorify martyrs, name streets, schools, and public squares after them, use foreign aid to fund terror, and reward convicted perpetrators with financial support exceeding the average employment rate. These actions are indicative of top-down policies that promote violence. From the pulpit to the public square, state-sponsored mass media, and authoritative religious sources, the voice of primary authority figures both reflects and inculcates collective ideology. The two main authoritative voices for extremist jihadist indoctrination today are Sunni Islamic theologian Yusuf al-Qaradawi of Egypt (now in Qatar) (see Table 2) and Ayatollah Khamenei, the Supreme Leader of theocratic Shia Iran (see Table 3).

**Table 2:** Top-down incitement examples: Yusuf al-Qaradawi

Date	Comment	Context
1995	“If everyone who defends his land and dies defending his sacred symbols is considered a terrorist, then I wish to be at the forefront of the terrorists. And I pray to Allah if that is terrorism, then O Allah make me live as a terrorist, die as a terrorist, and be raised up with the terrorists.” [81]	MAYA conference, Toledo, Ohio
3 Feb 2001	“He who commits suicide kills himself for his own benefit, while he who commits martyrdom sacrifices himself for the sake of his religion and his nation… He fights his enemy and the enemy of Allah with this new weapon, which destiny has put in the hands of the weak, so that they would fight against the evil of the strong and arrogant. The Mujahid becomes a ‘human bomb’ that blows up at a specific place and time, in the midst of the enemies of Allah and the homeland, leaving them helpless in the face of the brave Shahid.” [82]	Al-Ahram Al-Arabi newspaper (Egypt)
25 Apr 2001	“These operations are the supreme form of jihad for the sake of Allah, and a type of terrorism that is allowed by the Shari’a.” [82]	Al-Raya newspaper (Qatar)
July 2007	“I support the Palestinian cause. I support the resistance and the jihad. I support Hamas, the Islamic Jihad, and Hezbollah. I oppose the peace that Israel and America wish to dictate. This peace is an illusion. I support martyrdom operations.” [83]	Said at a conference held in his honor in Doha, Qatar
9 Jan 2009	“Take this oppressive, Jewish, Zionist band of people…do not spare a single one of them. Oh Allah, count their numbers, and kill them, down to the very last one.” [84]	Al Jazeera TV
12 Oct 2010	“Not one inch of the land of Islam must remain in the grasp of infidels and occupiers…We must irrigate the tree of freedom with our blood…. Arms must not be laid down—he who wants freedom must pay the price.” [85]	Al Aqsa Voice radio station
8 Dec 2017	“We make mistakes when we think that compromises might bring us honest solutions. Since the Arabs began negotiating with the Zionists, they have been giving up and giving up, until they almost gave up everything... The tongue cannot resist the tongue or resist the weapon by speaking. This is impossible. There must be resistance. This nation must resist and never surrender.” [86]	Tweet following President Trump’s recognition of Jerusalem, translated by Google Translate

**Table 3:** Top-down incitement examples: Ayatollah Khamenei

Date	Comment	Context
9 Mar 2015	“After negotiations, in Zionist regime they said they had no more concern about Iran for next 25 years; I’d say: Firstly, you will not see next 25 years; God willing, there will be nothing as Zionist regime by next 25 years. Secondly, until then, struggling, heroic and jihadi morale will leave no moment of serenity for Zionists.” [87]	Twitter—quote taken from a speech given earlier that day
29 Nov 2015	“The oppressed people of Palestine have experienced the worst kind of terrorism for the last sixty years… it is decades that a Palestinian family is not secure even in its own home from the Zionist regime’s death and destruction machinery. What kind of atrocious violence today is comparable to that of the settlement constructions of the Zionist regime?” [88]	Khamenei in an open letter to Western youth after the Paris terror attacks—Israel more ‘barbaric’ than Paris attackers
8 Feb 2016	“They (the US) support the child-killer Zionist regime and regional allies that are not familiar with and do not understand elections at all.” [89]	Khamenei in address to the Air Force and Air Defense commanders and personnel
14 Dec 2016	The Zionist regime -- as we have already said -- will cease to exist in the next 25 years if there is a collective and united struggle by the Palestinians and the Muslims against the Zionists.” [90]	Speaking during a meeting with the head of the Islamic Jihad terrorist group, Ramadan Abdullah Shalah.
21 Feb 2017	Khamenei described the Jewish state is a “fake nation” in a “dirty chapter of history that will be closed, with the grace of God,” a “cancerous tumor” that requires a “step by step” treatment. [91]	Speech at a pro-Palestinian gathering in Tehran.
7 Jul 2017	Ramadan terrorist bombings “are the outcome of nurturing terrorists by the security services of the US, the UK and the Zionist regime.” [92]	Speech for Eid-al Fitr at the conclusion of Ramadan
1 Jul 2014	“This rabid dog, this rapacious wolf, has attacked innocent people and humanity must show a reaction. This is genocide, a catastrophe of historical scale.” [93]	Khamenei during the Gaza Conflict in the summer of 2014
5 Aug 2015	“...planted this infected cancer gland within Islamic-Arabic territory. Today this gland has grown and become the cause of division among Muslim governments... Where their problems have come from? From this infected gland named “Israel” that was created by the superpowers.” [94]	Excerpt from Khamenei’s book “Palestine”.

#### Iran: Shiite theocracy

Ayatollah Khamenei has for many years promoted blatant dehumanization, delegitimization, and demonization of Jews, denying the Holocaust and referring to Jews as vermin, inhumane beasts and “sinister, unclean rabid dogs” and the State of Israel as a “cancerous tumor” and “infanticidal regime” doomed to utter ruin [53]. He has declared that the elimination of Israel is the central goal of the Islamic world today in order to eliminate its supposed crimes. Khamenei’s incitement and genocidal threats against Israel are now pandemic in much of the Muslim world and reflect the primary top-down authoritative voice of Shia ideology.

Textbooks in Iran require special attention, given the powerful state’s march towards nuclear capability. Iran is the world’s largest state exporter of terrorism, suppresses human rights in its own population, has the highest per-capita capital punishment rate in the world, and is an active enabler of Hezbollah (a recognized terror organization) and of the Syrian regime’s mass murder [54, 55]. Its leaders continue to demonize Israel and the West and consistently broadcast threats of destruction to Israel. The Iranian regime maintains the hardline to garner the support of its adherents and population and to deflect pressure and criticism of the regime’s shortcomings. These threats to destroy Israel are even more explicit than the veiled threats of Nazi Germany (note: the focus is on Israel not on the Jews—in order to shield themselves from the charge that they are engaging in Jew hatred.). Public rallies attended by top authority figures protest Israel’s right to exist and go as far as unveiling a digital countdown to its utter destruction [56]. This is still phrased as a deterministic prediction instead of an outright threat. However, Iran’s broad-scale indoctrination has to be taken seriously in light of its actions and its regime.

#### Saudi Arabia: center for Wahhabi doctrine

The Sunni Saudi Arabian government does not necessarily advocate extremist ideology and is rapidly changing under the new regime, making reforms concerning pluralism, women’s rights, and more. The question remains if they will succeed in these reforms without a complete overhaul of their educational system. Groiss has shown that Wahhabi textbooks project total enmity to Israel as a state and contain numerous examples of demonization and dehumanization of Jews invoking negative stereotypes and motifs taken from classic anti-Semitic propaganda [57]. The messages promoted through Saudi indoctrination are of concern because of the powerful status of Wahhabi theology in the Muslim world and the use of worldwide networks of mosques and schools to promote these messages [58].

Sunni and Shiite incitement has, since 1948, explicitly challenged and denied the right of the Jewish people to a homeland. The most insidious form of this delegitimization is to blatantly ignore Israel’s very existence. This form of indoctrination and incitement is difficult to study as it is invisible—a crime of omission rather than commission [59].

The deliberate and consistent omission of a people from history is an attempt to erase its very existence without force. Such delegitimization found in textbooks becomes intergenerational dogma and is a particularly insidious form of upstream indoctrination. An example of this omission is when Palestinian textbooks refer to peace based upon harmony between Christians and Muslims—without mentioning Jews.

#### Palestinian Authority

Indoctrination towards violence is replete from central figures in Palestinian society. Consider the implications of some of the primary tenets of the Hamas Charter: compulsory and eternal universal jihad, political and religious conflict with both Israel and the Jews aimed at their utter destruction; the uncompromising commitment to obtain all of Palestine; glorification of martyrs, etc. [60].

The Institute for Contemporary Affairs released a report indicating that, in 2017, a total of $344 million—representing 49.6% of all foreign aid funds received by the Palestinian Authority—was allocated to support terror (including lifelong salary stipends for convicted terrorists and families of “martyrs”) [61]. Such support incentivizes hate and builds intergenerational sustainability for terror.

Palestinian textbooks project many of the major motifs of Sunni indoctrination. This indoctrination is systemic and embedded but is expressed in incitement. Groiss, Shaked, and Pardo have extensively researched and scrutinized PA textbooks [62, 63]. The curriculum decidedly delegitimizes Israel, denies its past, includes rampant bias and disinformation, and glorifies acts of terror. The texts include less dehumanization than other textbooks, but they do promote perpetual war while ignoring the possibility of peace. Pardo delves into the radicalizing factors of both PA and Iranian curriculum and their influence [64].

Both Palestinian and Israeli textbooks have been widely scrutinized in light of the conflict. Such scrutiny has led to a reduction of dehumanizing metaphors in Palestinian textbooks but was not sensitive enough to discern invisible delegitimization of Israel and the Jews [46]. This observation indicates that such scrutiny is, in fact, effective in eliminating extreme forms of indoctrination, which supports the case for implementing specific population-wide countermeasures.

### Countermeasures

We suggest that the epidemiologic models presented can help us consider the relationship between indoctrination, incitement, and mass murder and help us to develop countermeasures to hateful ideological indoctrination in the light of current knowledge.

Classic modes of interventions for occupational and environmental exposures include surveillance and monitoring, removing or substituting hazardous exposures, introducing alternative messages, labeling, and screening. Fundamentally, there is also a need to redefine the “unacceptable” [65]. For example, we now realize in retrospect that population-wide exposure to lead, once considered normal, had adverse effects on mental health and development. Can we ask the same in regard to indoctrination and incitement? Background exposures once considered normal may need to be recognized as hazardous.

Benesch proposes a number of types of countermeasures that can be employed against dangerous speech (incitement and indoctrination) [35]. She distinguishes between traditional and alternative methods. Traditional methods consist of punitive measures and censorship. We call attention to cases of state-sanctioned indoctrination (notably, Iran). We include international sanctions and military force in the list of traditional countermeasures. These measures have shortcomings.

First, the use of censorship by governments raises ethical issues of privacy and free speech in democratic countries [13]. This tension was clearly evident throughout the process of drafting the Convention for the Prevention and Punishment of Genocide [66]. On the one hand, it is clear that governments and public health officials hold an ethical responsibility for their public’s health and to protect the community from harm. Tulchinsky dives into the history and development of community ethics and the challenges posed in balancing individual rights with public health. Failure to implement and enforce public health regulations could constitute negligence on the part of responsible parties [67]. Yet, we must recognize the unavoidable conflict between civil liberties and public security.

Second, there are instrumental issues with such measures. These traditional methods have a “limited and specific utility” limiting the spread and effects of specific pieces of incitement, while the influence of indoctrination and ideology remains [35]. Such ideologies are deeply embedded in the mindsets of affected populations. While these motifs may not be explicitly public, they can be triggered by key messages. In the digital age, with the mass dissemination of media messages through time and space, the text and subtext of incitement remains enduring and present in cyberspace. These can be forever retrievable by those programmed to look for them. For example, Twitter attempted to “clean out” ISIS-related incitement by banning hundreds of thousands of accounts. However, new accounts continue to open and many others have simply migrated to other social media sites [68].

Thus, the only way to definitively prevent future violence from the effects of incitement is to increase “audience resistance” [35]. The source of such audience susceptibility and receptiveness needs to be neutralized and countered. There is a need for population-wide measures to replace and counter hate with positive ideologies and to identify and strengthen positive role models. Benesch describes the successful case of a television drama created in Rwanda specifically to develop understanding and resistance to hate speech among the population [35].

The countermeasures proposed by Benesch may be successful in localized scenarios. However, we need to develop countermeasures to meet the challenges of systems of indoctrination that transcend traditional boundaries of space and time. Previous interventions that do not consider indoctrination may be inadequate. The current case studies proposed by Benesch are linked to election outcomes in Africa, triggered by a winner-takes-all election. They are successful, but may not be sufficient. Here, the stakes are much larger because they are global—as in the case of jihadi totalitarianism.

Sustainable strategies for prevention require population-wide strategies for removing the perpetrators of harmful ideology and the sources of incitement. The ideal (but not always practical) preventive strategy is to remove the source by defeating the perpetrators through military defeat. However, as in the case of Nazi Germany and Japan, military defeat was not sufficient in and of itself. Defeating the ideology of Nazism required a top-down comprehensive strategy to reeducate the entire population with values and motifs that replaced the old models and motifs, which had led them to support the regime. This was successfully implemented under the Allied Occupation and Marshall Plan in Europe, which included a massive overhaul of societal, economic, and educational programs [46].

Another innovative attempt at developing a countermeasure is Google’s “Redirect Method” to counter ISIS indoctrination and incitement online [69]. The project’s researchers identify vulnerable individuals searching for and viewing ISIS material. These individuals are then gradually and unobtrusively redirected to material that counters ISIS ideology, including Islamic criticism of the religious doctrines as well as evidence countering the public image of the organization’s economic success and popularity. This approach rejects censorship, instead seeking to address the deeper influence of indoctrination.

There is an oceanic depth of data available in the modern information age. Could search engines or media platforms be further developed to systematically probe the Internet for deep trends, analyzing what is happening in the sea of ideas, motifs, and messages (See Google “X” [70])? Just as people study sea or air pollution, similar concepts could be employed to probe word pollution. Such methods would not be preventive in nature but could identify trends for the sake of prediction and precaution. Tulchinsky describes the importance of international surveillance networks to monitor such word pollution [67].

Another effective countermeasure is to promote positive deviance and protect role models that embrace tolerance. Reinforcing the status and safety of positive role models is central to a strategy of ideological change [71]. Rather than paying terrorist salaries, what if authority figures supported those who exemplify positive deviance and moderation? There is a strong need for the world to be vigilant to ensure that foreign aid funds are not used to support terror.

Proposing precautionary countermeasures to identify dangerous and hateful ideology aims to advance the locus of detection to earlier stages. Any countermeasures would need to be measured in terms of their validity to determine if they measure what they purport to measure.

### Emerging issues and limitations

In this study, we presented only a modest number of examples of indoctrination as an upstream factor to genocide and genocidal terror. We recognize that several limitations apply to our study.

As noted above, epidemiology is guided by the basic questions: who, what, where, when, and how. The more difficult question to address may be why indoctrination and incitement exist in the first place. This question is beyond the scope of this paper yet deserves contemplation. This essay is not intended to probe what are the sources of evil or identify what are the sources of goodness. Because we recognize a cause-effect relationship between ideologies and actions, we have to ask what it is in belief systems that result in evil outcomes. We have not examined how religious, ethnic, or racial factors contribute to genocide or genocidal terror. Our premise is that ideology subsumes religion and crosses traditional, racial, and ethnic boundaries.

We have not dedicated adequate attention to emerging issues in Europe and the Far East, in particular, the burgeoning Rohingya crisis in Myanmar [72]. The Muslim Rohingya minority in Myanmar has been subjected to systematic top-down government-organized classification, symbolization, persecution, exclusion from citizenship, threats, atrocities, forced expulsion, and organized killing. A recent report released by Medecins Sans Frontieres (MSF) revealed that, according to conservative estimates, 647,000+ Rohingya have been forced to flee into Bangladesh, and 6700+ Rohingya have been killed since August 2017 [73–75]. The Rohingya are an example of a Muslim minority that has suffered discrimination, dehumanization, demonization, delegitimization, and genocidal crimes against humanity not resulting from totalitarian jihadist indoctrination or perpetration. Information is not readily available to the degree to which indoctrination and incitement were instrumental in this scenario. There is a need to examine whether our models apply in such settings today.

A major limitation of our models on indoctrination and incitement leading genocide is how to account for the governmental campaign of butchery and barbarity in Syria. There does not appear to be a history of incitement against those targeted by unspeakable atrocities and crimes against humanity perpetrated by Assad’s regime. We acknowledge that our models may not provide explanatory power for the crisis in Syria, in which 470,000+ people have been killed and more than 12 million have become refugees [76].

Our study does not delve into the reemergence of xenophobia and anti-Semitism in Europe, hate crimes in the United States, nor consider neo-nationalist and alt-right movements, all of which pose emerging threats today [77]. These social trends, potentially triggered by undercurrents of racism or the mounting immigration crisis and fear, bear alarming parallels to the development of fascist and Nazi movements in Europe in the 1930s, which made ample use of racist motifs and stereotypes to dehumanize and demonize minority groups. Problems posed by immigration may be real but are increasingly dangerous when they become a pretext for using the 5 Ds.

Due to the authoritarian nature of totalitarian regimes and societies, there is limited data regarding “hidden” populations (outliers/positive deviance) that reject the hateful motifs of extremist jihad and yet are silenced due to intimidation, coercion, force, or cultural restrictions. There may be many among these societies that, in fact, favor more moderate ideologies and yet remain anonymous out of fear.

In applying the concepts of epidemiology to the study of genocide prevention, we have to be aware of the dangers and potential abuses of proposing population-wide countermeasures to any system of belief or ethos (e.g., indoctrination of alternative motifs). Such countermeasures need to be weighed in regard to possible dissonance between civil liberties and basic human rights.

A potential criticism to our assertions is the idea that these concepts are largely theoretical as opposed to evidence-based. Statements about cause-effect relationships between incitement and violence may be questioned based upon ecologic associations [78]. Whenever there are temporal associations between increases in measures of incitement and increases in measures of actual violence or, conversely, decreases in both, these empirical associations provide plausible evidence for cause-effect relationships. When the population under observation is followed over time, then before-after comparisons control for confounders.

There is clear potential for population-wide intervention as demonstrated by the Rose Model. Specific interventions directed at groups rather than individuals (such as text messaging campaigns in Africa, the post-war educational reform in Europe and Japan after World War II) are examples of population-wide countermeasures that resulted in total change to political systems of indoctrination [79]. Their stories provide excellent empirical evidence for our argument. Such temporal associations are highly suggestive of true cause-effect relationships, even if the analysis is not brought from the macro to the micro. What we are looking for is a change in one group related to a change in another group over time—or, the delta versus the delta over time.

The examples presented are theoretical concepts with empirical validity that have explanatory power. Even so, actual epidemiological studies will need to be carried out to provide specific and additional evidence for our assertions, as well as quantify the relationship between variables. Gordon reiterates the premise that there is a cause-effect relationship between hate speech and mass atrocities including genocide and he proposes a unified legal approach to accountability for the consequences, in the context of international criminal law [80].

## Conclusion

The key message of this study is the need to recognize that ideologies and indoctrination are precursors to incitement to genocide and genocidal terror. In the twentieth and twenty-first centuries, ideologies of Nazism, Communism, and jihadist totalitarianism have had worldwide impact, bringing death and suffering to hundreds of millions. Jihadist totalitarianism is of particular concern today. The spread of its message—thanks to cyberspace and social media—transcends national and political borders, time, and space. Its influence and power is enhanced by theocratic dictatorial regimes, and its motifs are global in outreach. The scale of the threat is potentially larger in magnitude than previous genocides confined within national and political boundaries. Strategies and interventions need to recognize the unique challenges posed by this threat.

The world paid a heavy price for its failure to counter the vitriol of the aggressive genocidal Nazi ideology from the very outset. Jihadist indoctrination and incitement pose the possibility of similar dangers to today’s world unless they are counteracted before they result in the mass suffering of millions. The earlier one targets the source of population-wide exposure, the greater the benefits of success and the less costly the consequences. This is the case for proactive, preventive, and preemptive approaches to these threats.

The effects of jihadist and Islamist genocidal indoctrination and incitement are increasing. We suggest that the epidemiologic models presented provide a unified field theory for defining types and intensities of exposures, risks, and interventions. These models can enable us to predict what is (or what is not) achievable by countering the messages and motifs aimed at indoctrinating entire populations as well as direct effects on susceptible groups and individuals—in particular, the youth. It is not sufficient to simply search out and identify those at greatest risk for such messages and motifs. Counterstrategies must reach entire populations, promoting positive deviance, protecting and empowering good role models, and addressing the importance of top-down messages from authority figures. It is vital to recognize the role of educational systems in the intergenerational transmission of ideologies and, where necessary, initiate broad strategies for population-wide reeducation rooted in a value for life and human dignity. There is a need to propose and implement countermeasures such as new technologies for deep mining of data in cyberspace. The point of all these strategies is to keep with the Geoffrey Rose model of influencing entire populations towards greater health.

There is a need for policy makers, health care professionals, scholars, and world leaders to utilize their expertise and influence and take action, raising awareness and implementing policies and practices that challenge hateful ideologies and restore classic ideologies that emphasize respect for life and human dignity for all. These values, endorsed by the Universal Declaration of Human Rights, are core values of all the great universal faiths and should be basic to all belief systems. Counterstrategies must derive from the primacy of these universal core values. Otherwise, attempts to eliminate the messages and motifs in totalitarian jihadist ideologies that lead to genocidal terror in today’s world cannot be expected to succeed. Genocide, genocidal terror, and incitement result from human choice and bystander indifference. The timelines of all genocidal terror point to missed opportunities for intervention by bystanders of all kinds, to the degree that we now know the early warning signs, markers, and indicators of genocide. Intervention by bystanders has to be triggered by early warning signs and not by the outcomes, which, by that time, is too late. This observation has been the case for our invoking the Precautionary Principle in genocide prevention. The world can eradicate these horrific outcomes by reinforcing a policy of zero-tolerance for incitement and indoctrination of ideologies that lead to genocide and genocidal terror.

## Abbreviations

EW: Early warning
ICC: International Criminal Court
ISIS (Daesh): Islamic State in Iraq and Syria
MSF: Medecins Sans Frontieres
PA: Palestinian Authority
RA: Risk assessment
The 5 Ds of incitement: Dehumanization, demonization, delegitimization, disinformation, and denial of past atrocities
UN: United Nations
US: United States
